# Xuan Fei Bai Du Fang for treating coronavirus disease 2019: A protocol for systematic review and meta-analysis

**DOI:** 10.1097/MD.0000000000023485

**Published:** 2021-01-08

**Authors:** Yuying Cui, Jinming Yao, Guoliang Shao, Lin Liao

**Affiliations:** a Master of Clinical Integration in Traditional Chinese and Western Medicine, College of Traditional Chinese Medicine, Shandong University of Traditional Chinese Medicine, Ji-nan; b Department of Endocrinology and Metabology, First Affiliated Hospital of Shandong First Medical University; c Department of Endocrinology, Shandong University of Traditional Chinese Medicine Affiliated Hospital; d Acupuncture and Tuina College, Shandong University of Traditional Chinese Medicine, Jinan, China. Department of Endocrinology, Shandong University Qilu Hospital, Ji-nan 250014, China.

**Keywords:** coronavirus disease 2019, meta-analysis, protocol, systematic review, Xuan Fei Bai Du Fang

## Abstract

**Background:**

Coronavirus disease 2019 (COVID-19) is a respiratory infectious disease with a high fatality rate. Up to now, there are an estimated 26 million confirmed cases and 865,000 deaths around the world. But no effective way can control this disease. As the country that first discovered and treated the COVID-19, China has formed relatively mature prevention and treatment methods such as “3 prescriptions and 3 drugs.” Xuan Fei Bai Du Fang, as a member of “3 prescriptions and 3 drugs,” has very good clinical effects

**Methods:**

The PubMed, EMBASE, ClinicalTrials.gov, Cochrane Library, China National Knowledge Infrastructure, and Wanfang databases were searched for randomized controlled studies published to date. This study only screens clinical randomized controlled trials on QFBDF for COVID-19 to evaluate its efficacy and safety.

Import all literatures that meet the requirements into Endnote X9 software. The information was finally cross-checked by 2 reviewers. Papers selected for review were assessed for risk of bias according to the criteria. Quality assessment on design of study, risk of bias, indirectness and imprecision were assessed using the GRADE framework. Where sufficient studies were available, publication bias was assessed visually using funnel plots. Relative risks for primary and secondary outcomes were calculated on an intent-to-treat basis and pooled using random effects meta-analysis. the continuous is expressed by mean difference or standard mean difference, eventually the data is synthesized using a fixed effect model or a random effect model depending on whether or not heterogeneity exists. The heterogeneity of studies will be evaluated by *Q*-test and *I*^2^ statistic with RevMan5.3.

**Results:**

The time from a positive diagnosis to a negative result of 2 consecutive nucleic acid tests (not on the same day), cure rate. The results of our research will be published in a peer-reviewed journal.

**Conclusion:**

The purpose of this systematic review is to provide new evidence for the effectiveness and safety of Xuan Fei Bai Du Fang in the treatment of COVID-19.

**PROSPERO registration number:**

CRD42020213950.

## 1 Introduction

The World Health Organization (WHO) announced on March 11th 2020, that the outbreak of “Corona Virus Disease 20 19” (COVID-19), which initially started in Asia, has become pandemic. On September 4^th^ 2020 the etiologic agent “Severe Acute Respiratory Syndrome (SARS)-CoV-2 has spread all over the world with a global estimation of around 26 million confirmed cases and around 865,000 deaths.^[1]^ This disease is characterized by being highly contagious, and people of all ages are susceptible to infection. WHO. As of September 30, 2020, more than 30,000,000 confirmed COVID-19 cases have been reported to the World Health Organization, including more than 1,000,000 deaths. According to the conclusion drawn by the World Health Organization (WHO), there is no effective way to control the COVID-19.^[2]^ It is reported that the containment measures implemented in China have (at least for the moment) reduced new cases by more than 90%.^[3]^ Xuan Fei Bai Du Fang (XFBDF) is 1 of the 3 most important prescriptions in fighting the epidemic, and it is widely used in the treatment of patients. This recipe consists of 13 Chinese medicines, including Ephedrae Herba (Mahuang), Armeniacae Semen Amarum (Xingren), Raw Gypsum (Shigao), Semen Coicis (Yiyiren), Atractylodis Rhizoma (Cangzhu), Pogostemonis Herba (Guang Huoxiang), Artemisia annua (Qinghao), Polygoni Cuspidati Rhizoma et Radix (Huzhang), Verbena officinalis (Mabiancao) 30 g, Phragmitis rhizome (Lugen) 30 g, Semen Lepidii Apetali (Tinglizi) 15 g, Citri Exocarpium Rubrum (Juhong) 15 g, Glycyrrhizae Radix Et Rhizoma (Gancao) 10 g. It has the effects of dispelling the lungs, dispelling dampness, clearing away heat and evils, purging the lungs and detoxification, and is suitable for mild and common COVID-19 patients. Clinical data shows that this prescription has obvious effects in shortening the disappearance of clinical symptoms of COVID-19, the time to return to normal body temperature, and the average length of stay in hospital. It also shows unique advantages in blocking the transition from mild to severe.^[4]^

Therefore, we aim to collect all randomized controlled trials related to XFBDF in the treatment of COVID-19, and conduct meta-analysis and systematic reviews to provide evidence-based medicine for the treatment of COVID-19.

## 2 Methods

### 2.1 Protocol registration

This systematic review protocol has been registered in the PROSPERO network (No. CRD42020213950). We used the Preferred Reporting Items for Systematic Reviews and Meta-Analyses Statement^[5]^ guidelines in this study. As this was a review of published literature, ethics committee approval and patient consent were not required. We will update our protocol for any changes in the entire research process if needed.

### 2.2 Inclusion criteria

#### 2.2.1 Criteria for considering studies for this review types of studies

This review considers both experimental and epidemiological study designs of XFBDF for COVID-19, including randomized controlled trials (RCTs), non-randomized controlled trials, before and after studies, prospective, retrospective and comparative cohort studies, and analytical cross-sectional studies for inclusion.

#### 2.2.2 Types of participants

This study included patients who had been clearly diagnosed with the new coronavirus disease. Except that participants must be over 18 years old, there were no strict restrictions on gender and severity of the disease.

#### 2.2.3 Type of interventions

The test group uses XFBDF. The control group can be treated with other treatments except XFBDF. There are no obvious restrictions on the dosage of therapeutic drugs and specific intervention routes.

#### 2.2.4 Types of outcome measures

Total clinical effective rate, improvement rate of lung CT, adverse events.

### 2.3 Search strategy

A preliminary search of PubMed and Embase was undertaken to identify key text words contained in the titles and abstracts of relevant articles, and of the index terms used to describe an article. A second search, using all identified keywords and index terms, was then undertaken across the following databases: PubMed, EMBASE and Cochrane Central Register of Controlled Trials (Central), China National Knowledge Infrastructure, Wan-fang database, Chinese Scientific Journal Database, Chinese Biomedical Literature Databases. Exemplary search strategy of PubMed is listed in Table 1.

**Table 1:** PubMed search strategy.

Number	Search terms
1	“COVID-19” [Title/Abstract] OR “COVID19” [Title/Abstract] OR “coronavirus disease 2019” [Title/Abstract]OR “coronavirus disease-19” [Title/Abstract] OR“2019 novel coronavirus disease” [Title/Abstract] OR “2019-nCoV disease” [Title/Abstract] OR “2019-nCoV infection” [Title/Abstract] OR “2019-nCoV ” [Title/Abstract] OR “SARSCov2 ” [Title/Abstract] OR “SARS-CoV ” [Title/Abstract] OR “2019 coronavirus ” [Title/Abstract] OR “2019 novel coronavirus infection” [Title/Abstract]
2	“Xuanfeibaidufang” [All Fields] OR “Xuan Fei Bai Du Fang” [All Fields] OR “Xuanfeibaidu Decoction” [All Fields]OR “Xuan Fei Bai Du Decoction” [All Fields] OR “XFBD” [All Fields] OR “XFBD Fang” [All Fields]
3	“randomized controlled trial” [Publication Type] OR “controlled clinical trial” [Publication Type] OR “Single-Blind Method” [Text Word] OR “random allocation” [Text Word] OR “allocation” [Text Word] OR “RCT” [Text Word]OR “RCTs” [Text Word]
4	#1 AND #2 AND #3

### 2.4 Data collection and analysis

#### 2.4.1 Selection of studies

**Figure 1:**
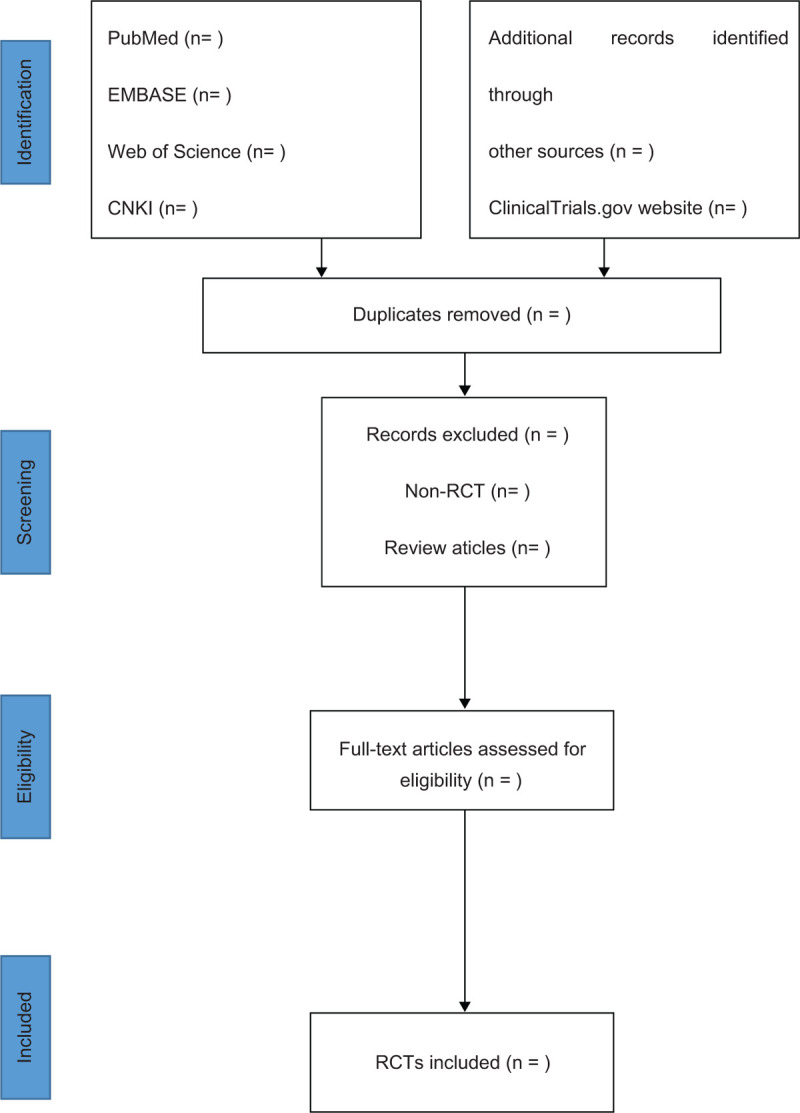
Study flowchart of selected articles for final analysis.

Import all literatures that meet the requirements into Endnote X9 software. Two reviewers (GLS and YYC) will independently select the studies. They will check the results with each other. When disagreements occur, a third reviewer (JMY) will make the final decision. They will read the full texts of all included studies if necessary. Screening operation will flow the diagram of Figure 1. If the full literatures are unable to be obtained or related data are incomplete, we will contact the corresponding author.

#### 2.4.2 Data extraction and management

According to the characteristics of the study, we prepare an excel form for data collection before data extraction. Outcome indicators for eligible studies were independently extracted and filled in the data extraction form by 2 reviewers. The main data extracted are as follows: title, author, year, fund source, sample size, age, sex, duration of disease, interventions, outcome measures, adverse reactions, etc. If there are something unclear, you can not hesitate to contact authors of more detailed information. The above information was finally cross-checked by 2 reviewers.

#### 2.4.3 Data extraction and analysis

Papers selected for review were assessed for risk of bias according to the following criteria: random sequence generation (selection bias), allocation concealment (selection bias), blinding of participants and personnel (performance bias), blinding of outcome assessment (detection bias), selective reporting (reporting bias), comparability of baseline groups, application of intent-to-treat analysis, and proportion lost-to follow up. Quality assessment on design of study, risk of bias, inconsistency, indirectness and imprecision were assessed using the GRADE framework.^[6]^ Where sufficient studies were available, publication bias was assessed visually using funnel plots. Relative risks for primary and secondary outcomes were calculated on an intent-to-treat basis and pooled using random effects meta-analysis. Where statistical pooling was not possible or deemed inappropriate, study-specific outcomes are presented. The heterogeneity of studies will be evaluated by *Q*-test and I2statistic with RevMan5.3. Heterogeneity was examined using the *x*^2^ statistic with a significance level of.0.10, and the *I*^2^ statistic with an I2estimate greater than 50% was considered indicative of moderate to high levels of heterogeneity.^[7]^

## 3 Discussion

Due to the high fatality rate and high infectiousness of COVID-19, it has caused a pandemic worldwide.^[8,9]^ As the northern hemisphere enters winter, the second wave of epidemics is making a comeback. At present, apart from antiviral therapy (such as cocktail therapy)^[10]^ and symptomatic treatment, no unified treatment method has been established.^[11]^ Although countries are trying their best to speed up the development of vaccines, there are currently 10 vaccines in Phase III clinical trials and cannot be put into use on a large scale, according to statistics from the World Health Organization^[1]^

Traditional Chinese medicine has shown good clinical effects in the treatment of COVID-19 with fewer side effects.^[12]^ Studies have confirmed that Qing Fei Pai Du Fang, the first of the 3 anti-epidemic prescriptions, has immune regulation, anti-infection, anti-inflammatory and multi-organ protection effects in the treatment of COVID-19.^[1]^ We aim to study the effectiveness and safety of XFBDF, 1 of the 3 anti-epidemic prescriptions, in the treatment of COVID-19, and to provide a more extensive and effective treatment method for the upcoming second wave of epidemic climax.

## Author contributions

**Conceptualization:** Yuying Cui.

**Data curation:** Yuying Cui.

**Formal analysis:** Yuying Cui, Lin Liao.

**Funding acquisition:** Lin Liao.

**Investigation:** Yuying Cui, Jinming Yao.

**Methodology:** Yuying Cui, Jinming Yao, Lin Liao.

**Project administration:** Jinming Yao, Lin Liao.

**Resources:** Jinming Yao, Lin Liao.

**Software:** Jinming Yao, Guoliang Shao.

**Supervision:** Guoliang Shao.

**Validation:** Guoliang Shao, Lin Liao.

**Visualization:** Guoliang Shao.

**Writing – original draft:** Guoliang Shao.

**Writing – review & editing:** Guoliang Shao, Lin Liao.
